# Supplementary material for: Genome-wide association study finds multiple loci associated with intraocular pressure in HS rats
Source: Front Genet. 2023 Jan 30;13:1029058. doi: 10.3389/fgene.2022.1029058 (PMC9922724; doi:10.3389/fgene.2022.1029058)
Supplement: Supplementary file 1 [file DataSheet1.PDF]

Supplemental materials.

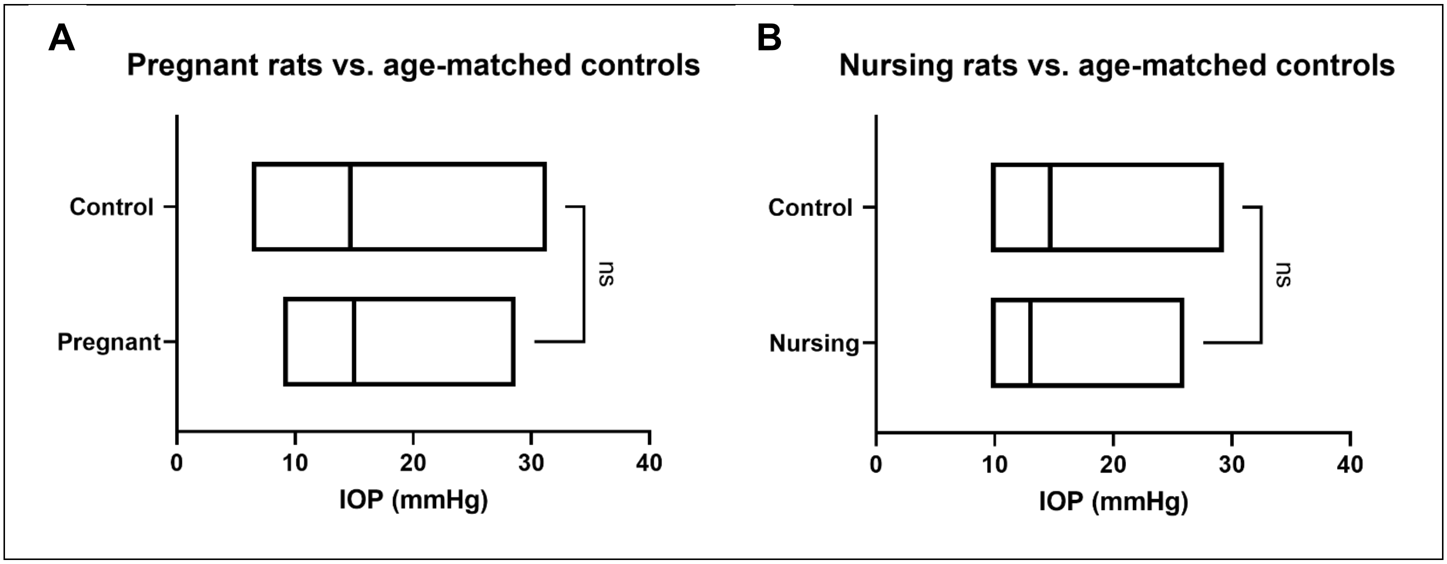

Supplemental Figure 1. Boxplot demonstrating IOP values from age-matched control rats vs. pregnant (n = 86) and nursing (n = 26) rats. (**A**) IOP of pregnant rats and age-matched controls. (**B**) IOP of nursing rats and age-matched controls. Number of control rats was equivalent to respective pregnant and nursing rats groups. Compared using Mann-Whitney test ($p < 0.05$). ns = non-significant. Line shows the mean.

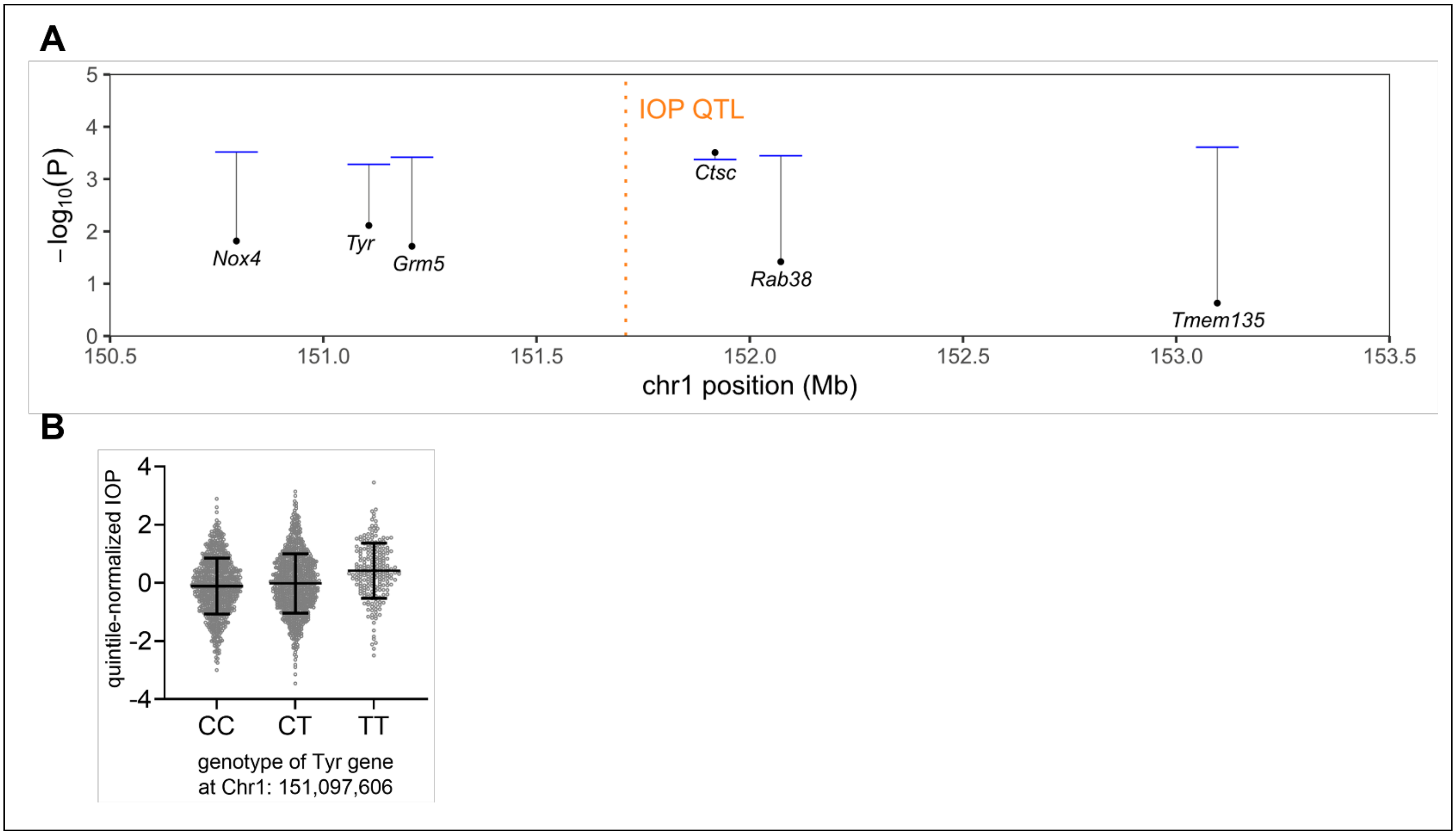

Supplemental Figure 2. Additional information on the QTL on Chromosome 1. (**A**) Association between gene expression and genotypes in the QTL on chromosome 1. The top-associated cis-window (+/- 0.5 Mb) SNP for expression levels of each gene within this range is indicated as a point. Each gene-specific significance threshold, determined by permutations of the gene's cis-window data, is shown as a horizontal segment connected to its top-SNP point so that a point lying below its segment indicates no significant cis-eQTL was found for that gene from whole eye tissue. (**B**) The effect plot for the *Tyr* genotype at chr1:151,097,606 shows IOP in rats with three genotypes where variant TT leads to dysfunctional protein tyrosinase and albinism in rats. The IOP is not statistically significantly different for 3 genotypes (One-way ANOVA followed by Brown-Forsythe posthoc test, $p = 0.0825$). IOP is shown as residuals after normalization performed as described in the "Methods" section. The number of individuals with each genotype: 726 (TT), 855 (CT), and 213 (TT).

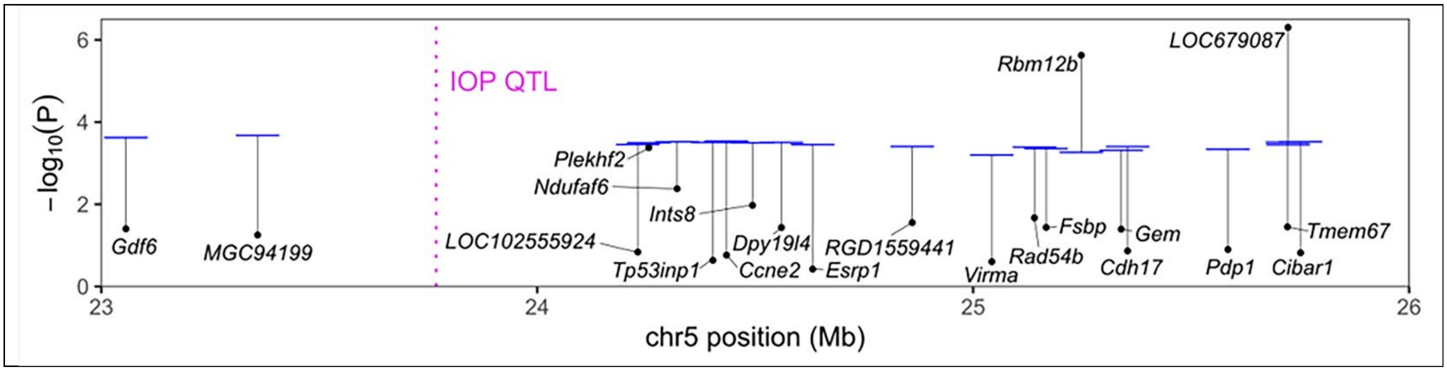

Supplemental Figure 3. Additional information on QTL on Chromosome 5. Association between gene expression and genotypes in the QTL on chromosome 1. The top-associated cis-window (+/- 0.5 Mb) SNP for expression levels of each gene within the 23-26 Mb range is shown as a point. Each gene-specific significance threshold, determined by permutations of the cis-window data of each gene, is shown as a horizontal segment connected to its top-SNP point, so that a point lying below its segment indicates no significant cis-eQTL was found for that gene from whole eye tissue. While both *Rbm12b* and L*OC679087* were cis-eQTLs, they are located outside of the IOP QTL region and not in LD with the QTL's top SNP. r2 between the top SNP of IOP (chr5:23768259) with the top SNPs of *Rbm12b* and *LOC679087* top (chr5:25600721 and chr5:25437533) are 0.397 and 0.395, respectively. Association analysis for *Plekhf2* expression shows that nominal *p*-value for cis-eQTL is close to the threshold.

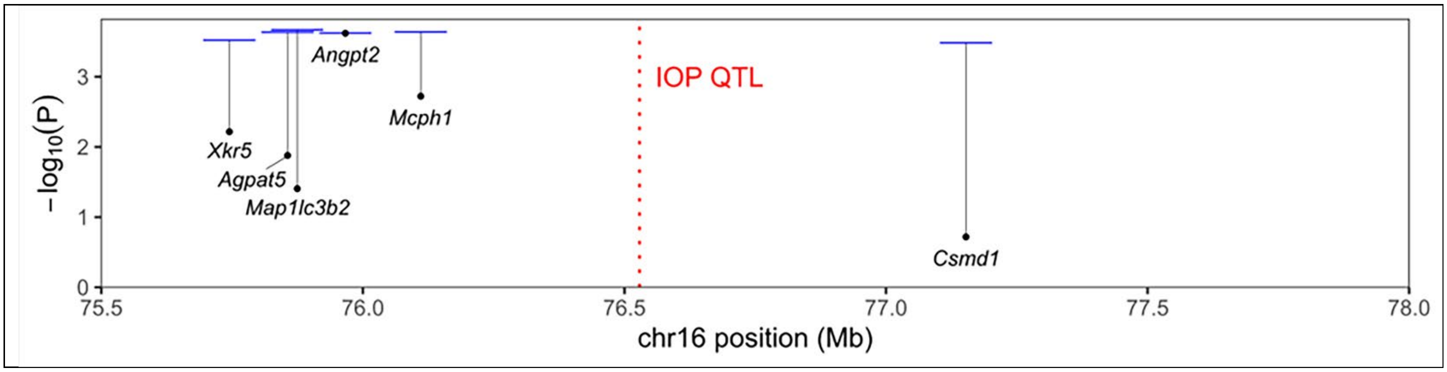

Supplemental Figure 4. Association between gene expression and genotypes in the QTL on chromosome 16. The top-associated cis-window (+/- 0.5 Mb) SNP for expression levels of each gene within the QTL range is shown as a point. Each gene-specific significance threshold, determined by permutations of the gene's cis-window data, is shown as a horizontal segment connected to its top-SNP point, so that a point lying below its segment indicates no significant cis-eQTL was found for that gene from whole eye tissue. The p-value for the *Angpt2* eQTL is at a threshold for significance.

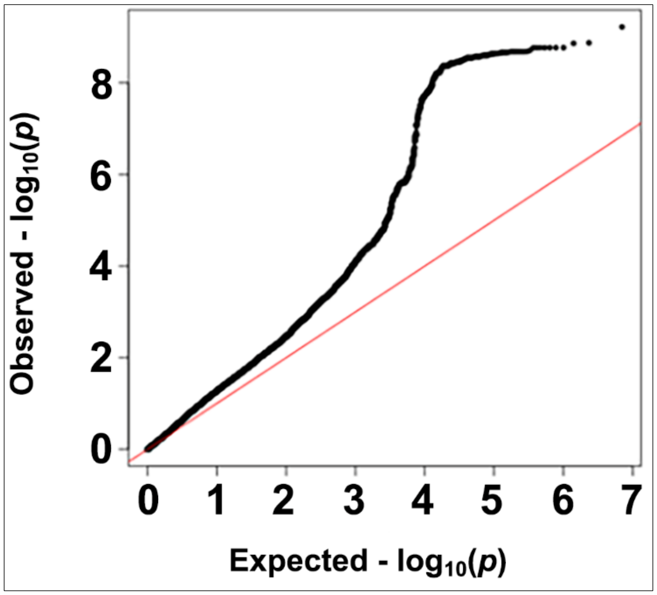

Supplemental Figure 5. The QQ plot for IOP GWAS.
